# Crystal structures of 2,4,6-tri­iodo­benzo­nitrile and 2,4,6-tri­iodo­phenyl isocyanide

**DOI:** 10.1107/S2056989017018217

**Published:** 2018-01-09

**Authors:** Wayland E. Noland, Doyle Britton, Gregory K. Sutton, Andrew K. Schneerer, Kenneth J. Tritch

**Affiliations:** a Department of Chemistry, University of Minnesota, 207 Pleasant St SE, Minneapolis, MN 55455, USA

**Keywords:** crystal structure, nitrile, isocyanide, N⋯I contacts, C⋯I contacts

## Abstract

The title crystals, C_7_H_2_I_3_N, are isomorphous. Both mol­ecules lie across two crystallographic mirror planes and a twofold axis. The principal supra­molecular inter­action is centric *R*_2_^2^(10) CN/NC⋯I short contacts involving both *ortho* I atoms, with two contacts bis­ecting each cyano and iso­cyano group. These form ribbons along [010] and give rise to a planar sheet structure parallel to (100). All pairs of adjacent sheets have centric stacking, a mode not previously reported for sheets of this type. This study completes the series of *homo*-2,4,6-trihalobenzo­nitriles, in which I atoms give the strongest CN⋯*X* and NC⋯*X* inter­actions (*X* = F, Cl, Br, I).

## Abstract 2

The title crystals are isomorphous, and form centrically stacked planar sheets formed by CN⋯I and NC⋯I short contacts.

## Chemical context   

**Figure 1:**
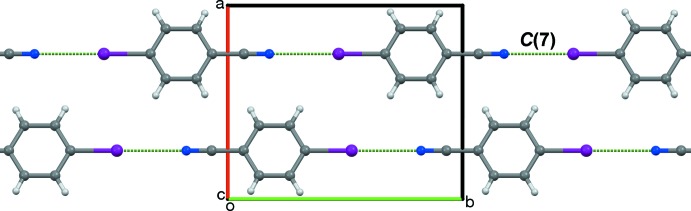
Several mol­ecules in the crystal of 4-iodo­benzo­nitrile (4ICN), viewed along [001]. Dashed green lines represent CN⋯I short contacts, which collectively form a *C*(7) chain motif along [010]. All previously reported 4-iodo­benzo­nitriles form similar chains.

**Figure 2:**
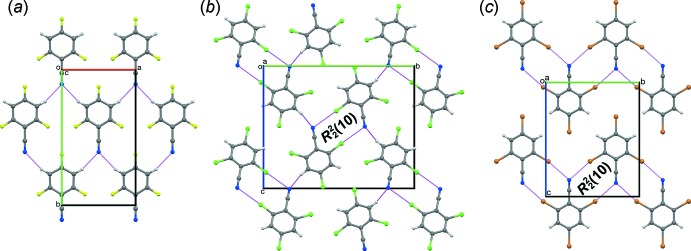
(*a*) A sheet in a crystal of F3CN, showing two CN⋯H contacts per CN group, viewed along [001]; (*b*) A sheet in a crystal of Cl3CN, showing one CN⋯H and one CN⋯Cl contact per CN group, viewed along [100]; (*c*) A sheet in the *Z* = 8 polytype of Br3CN, showing two CN⋯Br contacts per CN group, viewed along [100]. Dashed magenta lines represent short contacts.

The strength of cyano–halo inter­actions tends to increase with increasing polarizability, or the elemental period, of the halogen. Structure-directing CN⋯F inter­actions are usually not observed (Bond *et al.*, 2001 ▸). In crystals of the other 4-halobenzo­nitriles (*X* = Cl, Br, I), parallel or anti­parallel 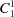
(7) CN⋯*X* chains dominate the secondary structures (Fig. 1 ▸; Desiraju & Harlow, 1989 ▸). When the halo atom is moved to the 2-position, 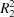
(10) CN⋯*X* rings can form, usually as inversion dimers. Halogenation at both *ortho* positions allows the formation of CN⋯*X*-derived ribbons or sheets. The aforementioned periodic trend is exhibited by the *homo*-2,4,6-trihalobenzo­nitriles. No CN⋯F contacts are observed in 2,4,6-tri­fluoro­benzo­nitrile (F3CN). Instead, each CN group is bis­ected by two CN⋯H contacts (Fig. 2 ▸ *a*; Britton, 2008 ▸). In 2,4,6-tri­chloro­benzo­nitrile (Cl3CN), half of these have been replaced by CN⋯Cl contacts (Fig. 2 ▸ *b*; Pink *et al.*, 2000 ▸). In 2,4,6-tri­bromo­benzo­nitrile (Br3CN), no CN⋯H contacts are found, and each CN group is bis­ected by two CN⋯Br contacts (Fig. 2 ▸ *c*; Britton *et al.*, 2016 ▸).
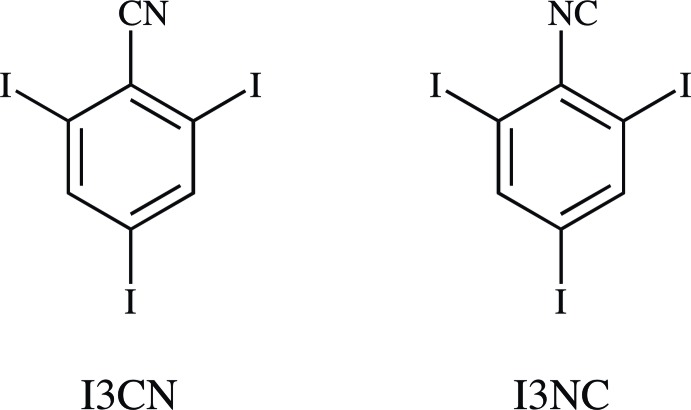


## Database survey   

No entries were found in the most recent update of the Cambridge Structural Database (Version 5.37, May 2017; Groom *et al.*, 2016 ▸) that have I atoms at both *ortho* positions of a benzo­nitrile. Four of the five crystalline 2-iodo­benzo­nitriles have CN⋯I contacts (Britton, 2001 ▸, 2004 ▸; Ketels *et al.*, 2017 ▸; Lam & Britton, 1974 ▸); the fifth is a cyano alcohol that forms O—H⋯NC hydrogen bonds (Salvati *et al.*, 2008 ▸). The 3-iodo analogs do not pack as efficiently. Three of the four examples feature I⋯I contacts (Britton, 2006 ▸; Merz, 2006 ▸); packing in the fourth example is directed by hydrogen bonding between acetamido groups (Garden *et al.*, 2007 ▸). All five reported 4-iodo­benzo­nitriles form *C*(7) CN⋯I chains (Fig. 1 ▸; Bond *et al.*, 2001 ▸; Britton, 2004 ▸; Desiraju & Harlow, 1989 ▸; Gleason & Britton, 1978 ▸). It is pertinent to determine the crystal structure of 2,4,6-tri­iodo­benzo­nitrile (I3CN) to complete the series of *homo*-2,4,6-trihalobenzo­nitriles, and to determine whether the primary packing inter­action is CN⋯I-derived *C*(7) chains, 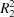
(10) rings, or another motif. 2,4,6-Tri­iodo­phenyl isocyanide (I3NC) is included to contribute to the library of corres­ponding halogenated nitrile-isocyanide crystal pairs.

## Structural commentary   

**Figure 3:**
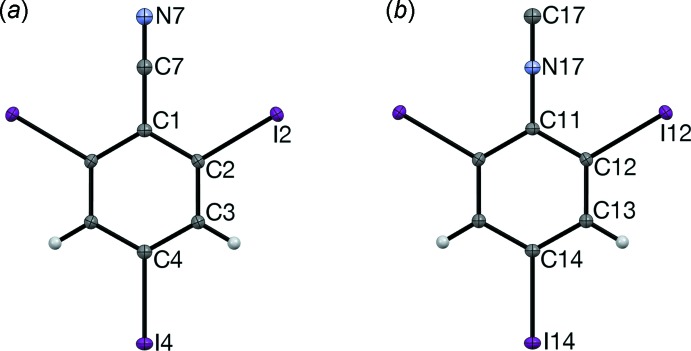
The mol­ecular structures of (*a*) I3CN and (*b*) I3NC, with atom labeling and displacement ellipsoids at the 50% probability level. Unlabeled atoms are generated by the symmetry operation (1 − *x*, 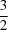 − *y*, *z*).

Mol­ecules of I3CN and I3NC (Fig. 3 ▸) lie about a twofold axis and two orthogonal vertical mirror planes. Thus, they have crystallographically-imposed *C_2v_* symmetry and are planar, with the *para* I atom (I4; I14) collinear with the CN and NC groups. All of the aryl bond angles are roughly 120°. The *ortho* I atoms (I2, I2′; I12, I12′) are scissored slightly toward the *ipso* C atom (C1; C11), which is probably caused by the inter­molecular CN⋯I and NC⋯I short contacts. The bond lengths are typical for their respective functional groups.

## Supra­molecular features   

**Figure 4:**
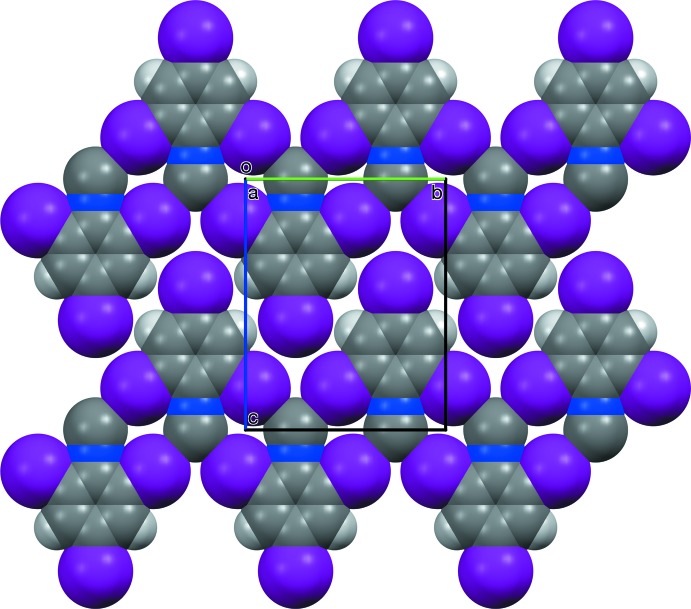
A space-filling drawing of the sheet structure of I3NC, viewed along [100].

**Figure 5:**
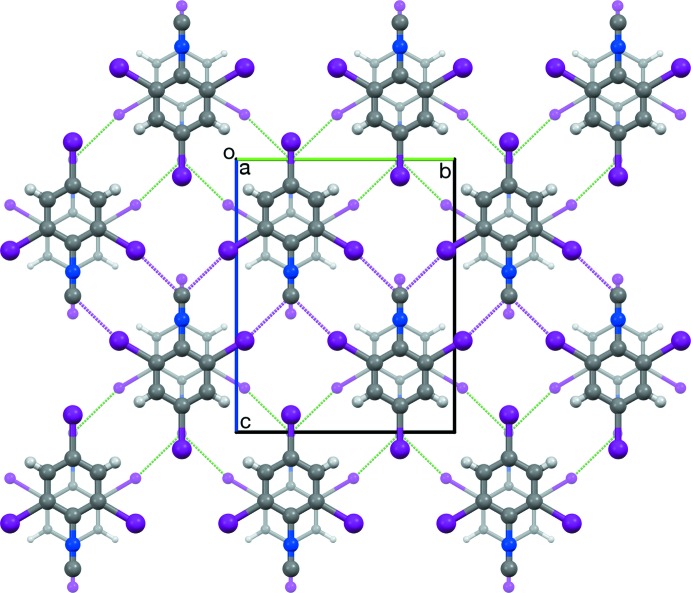
Two adjacent sheets in I3NC, viewed along [100], illustrating the centric stacking mode. Dashed magenta lines represent short contacts in the front layer. Mol­ecules in the rear layer are drawn with smaller balls and sticks, lower opacity, and green dashed lines representing short contacts.

Crystals of I3CN and I3NC are isomorphous. The CN and NC groups are bis­ected by C7≡N7⋯I2 and N17≡C17⋯I12 contacts (Table 1 ▸), forming ribbons of 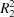
(10) rings parallel to (100) along [010]. Adjacent ribbons translate along [001]. The resulting planar sheet structure (Fig. 4 ▸) matches that observed in Br3CN and the corresponding isocyanide (Br3NC) (Britton *et al.*, 2016 ▸), and the 4-chloro (Britton, 2005 ▸) and 4-nitro (Noland & Tritch, 2017 ▸) analogs of Br3CN. In crystals of I3CN and I3NC, all pairs of adjacent sheets have centric stacking along [100] (Fig. 5 ▸), with mol­ecules stacked about a glide plane and an inversion center. In the polytypes of Br3CN and Br3NC, adjacent sheets had combinations of centric and translational stacking, but not solely centric stacking. The 4-chloro analog had translational stacking. The 4-nitro analog had glide stacking, with no inversion center between stacked mol­ecules. Thus, the all-centric stacking of I3CN and I3NC can be regarded as a new polytype in this series.

The mean CN⋯*X* contact lengths can be compared for *X* = Cl, Br, and I (Table 2 ▸). For 4-chloro­benzo­nitrile (4ClCN), 4-bromo­benzo­nitrile (4BrCN), and 4-iodo­benzo­nitrile (4ICN) (Table 2 ▸, col. 2), the contact distance decreases with increasing halogen size, highlighting the increase in contact strength (Desiraju & Harlow, 1989 ▸). This trend is essentially mirrored among 2,4,6-trihalobenzo­nitriles (Table 2 ▸, col. 3), although the contact distance in I3CN is 0.01 Å larger than in Br3CN. The N⋯*X* non-bonded contact radii are listed (Table 2 ▸, col. 4; Rowland & Taylor, 1996 ▸). The ‘shortness’ of contacts in 2,4,6-trihalobenzo­nitriles is expressed as the ratios of contact radii to the respective contact distances (Table 2 ▸, col. 5). A similar comparison of NC⋯*X* contact lengths in the corresponding trihalo isocyanides also shows decreasing contact length with increasing halogen size (Table 3 ▸, col. 2). The NC⋯*X* contacts have slightly greater shortness (Table 3 ▸, col. 4) than the corresponding CN⋯*X* contacts. The N17≡C17⋯I12 contacts in I3NC are the strongest cyano/iso­cyano–halo inter­actions in this series.

## Synthesis and crystallization   

**2,4,6-Tri­iodo­aniline (I3NH2)**, adapted from the work of Jackson & Whitmore (1915 ▸): Aniline (1.0 mL) and hydro­chloric acid (0.7 *M*, 850 mL) were combined and stirred in a round-bottomed flask. Iodine monochloride (8.2 g) was placed in a separate flask and then warmed to 315 K. The two flasks were connected with a glass bridge. A slow stream of nitro­gen was passed through the headspace in the second vessel so that the iodine monochloride was gradually swept into the first vessel over 2–4 d. After the transfer was complete, the reaction mixture was neutralized with NaHCO_3_ solution, followed by reduction of excess iodine by washing with Na_2_S_2_O_3_ solution. Di­chloro­methane (approx. 100 mL) was added, with stirring, until nearly all solids had dissolved. The organic portion was filtered through silica gel (3 cm H × 4 cm D), and then the filter was washed with di­chloro­methane (3 × 20 mL). The filtrate was placed in a loosely-covered beaker. After most of the di­chloro­methane had evaporated, beige needles were collected by suction filtration (4.48 g, 89%). M.p. 459–460 K (lit. 459); ^1^H NMR (300 MHz, CDCl_3_) *δ* 7.864 (*s*, 2H), 4.658 (*s*, 2H); ^13^C NMR (75 MHz, (CD_3_)_2_SO) *δ* 147.0 (1C), 145.4 (2C), 83.0 (2C), 78.8 (1C); IR (KBr, cm^−1^) 3417, 3056, 2987, 1632, 1422, 1265, 741, 704; MS (EI, *m*/*z*) [M]^+^ calculated for C_6_H_4_I_3_N 470.7472, found 470.7470.

**Figure 6:**
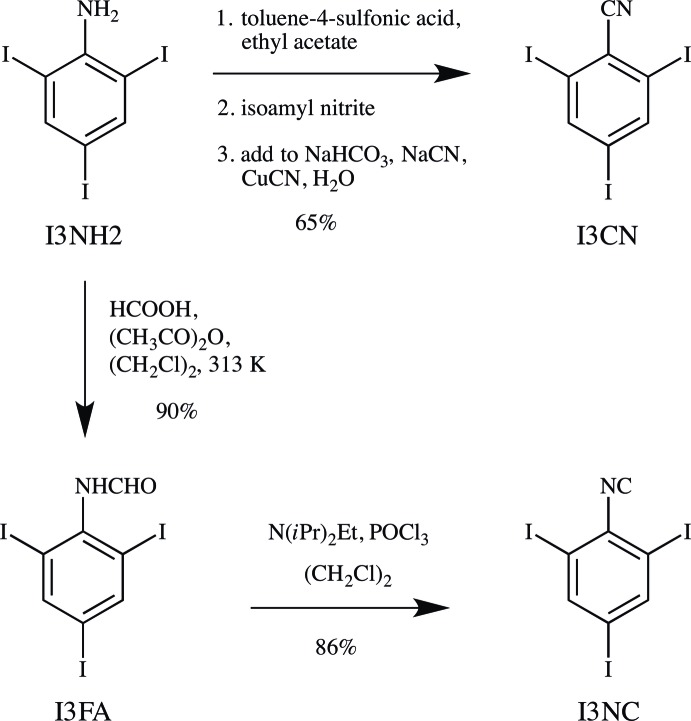
The synthesis of I3CN and I3NC.

**2,4,6-Tri­iodo­benzo­nitrile (I3CN)**, was prepared from I3NH2 (101 mg; Fig. 6 ▸) based on the Sandmeyer procedure described by Britton *et al.* (2016 ▸). Ethyl acetate (20 mL), toluene-4-sulfonic acid monohydrate (77 mg), and isoamyl nitrite (60 µL) were used in place of water, acetic and sulfuric acids, and sodium nitrite, respectively. The desired chromatographic fraction (*R_f_* = 0.44 in 4:1 hexa­ne–ethyl acetate) was concentrated on a rotary evaporator, giving a beige powder (67 mg, 65%). M.p. 517–518 K; ^1^H NMR (500 MHz, (CD_3_)_2_SO) *δ* 8.431 (*s*, 2H, H3); ^13^C NMR (126 MHz, CD_2_Cl_2_) *δ* 147.5 (2C, C3), 127.3 (1C, C1), 120.7 (1C, C7), 101.1 (1C, C4), 99.6 (2C, C2); IR (NaCl, cm^−1^) 3070, 2227, 1532, 1359, 1206, 1081, 861, 787, 706; MS (ESI, *m*/*z*) [*M* + Na]^+^ calculated for C_7_H_2_I_3_N 503.7213, found 503.7216.

**2,4,6-Tri­iodo­formanilide (I3FA)** was prepared from I3NH2 (1.01 g) according to the formyl­ation procedure described by Britton *et al.* (2016 ▸), with 1,2-di­chloro­ethane (10 mL and 100 mL) in place of tetra­hydro­furan, giving white needles (962 mg, 90%). M.p. 557–558 K; ^1^H NMR (300 MHz, (CD_3_)_2_SO; 2 conformers observed) *δ* 10.089 (*s*, 1H; major), 9.655 (*s*, 1H; minor), 8.303 (*s*, 1H; major), 8.278 (*s*, 2H; minor), 8.233 (*s*, 2H; major), 7.978 (*s*, 1H; minor); ^13^C NMR (126 MHz, (CD_3_)_2_SO; 2 conformers observed) *δ* 164.4 (1C; minor), 159.4 (1C; major), 146.4 (2C; minor), 146.1 (2C; major), 141.0 (1C; major), 140.4 (1C; minor), 102.4 (2C; minor), 101.9 (2C; major), 95.9 (1C; minor), 95.6 (1C; major); IR (NaCl, cm^−1^) 3221, 3076, 2919, 1637, 1490, 1380, 1232, 1143, 857, 794, 703, 682; MS (ESI, *m*/*z*) [M - H]^−^ calculated for C_7_H_4_I_3_NO 497.7354, found 497.7365.

**2,4,6-Tri­iodo­phenyl isocyanide (I3NC)** was prepared from I3FA (397 mg) according to the dehydration procedure described by Britton *et al.* (2016 ▸), giving a white powder (330 mg, 86%). M.p. 467–468 K; ^1^H NMR (300 MHz, CDCl_3_) 8.198 (*s*, 2H, H13); ^13^C NMR (126 MHz, (CD_3_)_2_SO) *δ* 170.0 (1C, C17), 146.2 (2C, C13), 133.8 (1C, C11), 98.8 (1C, C14), 97.7 (2C, C12); IR (KBr, cm^−1^) 3073, 3037, 2920, 2126, 1529, 1079, 861, 704; MS (ESI, *m*/*z*) [*M* - H]^−^ calculated for C_7_H_2_I_3_N 479.7249, found 479.7226.

**Crystallization:** Crystals of I3CN and I3NC were prepared by slow evaporation of aceto­nitrile solutions at ambient temperature, followed by deca­ntation and then washing with pentane.

## Refinement   

Crystal data, data collection and structure refinement details are summarized in Table 4 ▸. A direct-methods solution was calculated, followed by full-matrix least squares/difference-Fourier cycles. All H atoms were placed in calculated positions (C—H = 0.95 Å) and refined as riding atoms with *U*_iso_(H) set to 1.2*U*_eq_(C).

## Supplementary Material

Crystal structure: contains datablock(s) I3CN, I3NC. DOI: 10.1107/S2056989017018217/lh5864sup1.cif

Structure factors: contains datablock(s) I3CN. DOI: 10.1107/S2056989017018217/lh5864I3CNsup2.hkl

Structure factors: contains datablock(s) I3NC. DOI: 10.1107/S2056989017018217/lh5864I3NCsup3.hkl

Supporting information file. DOI: 10.1107/S2056989017018217/lh5864I3CNsup4.cml

Supporting information file. DOI: 10.1107/S2056989017018217/lh5864I3NCsup5.cml

CCDC references: 1580006, 1581218

Additional supporting information:  crystallographic information; 3D view; checkCIF report

## Figures and Tables

**Table 1:** Contact geometry (Å, °) for I3CN and I3NC

*A*≡*B*⋯I	*A*≡*B*	*B*⋯I	*A*≡*B*⋯I
C7≡N7⋯I2^i^	1.151 (3)	3.074 (2)	132.85 (3)
N17≡C17⋯I12^i^	1.164 (3)	3.106 (2)	134.18 (3)

Symmetry code: (i) −*x* + 1, *y* − 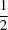, −*z* + 2

**Table 2:** Mean CN⋯*X* contact lengths (Å) in 4-halobenzo­nitriles (4*X*CN) and 2,4,6-trihalobenzo­nitriles (*X*3CN)

*X*	4*X*CN	*X*3CN	*r* [N + *X*] (Å)	[*r*/*X*3CN]
Cl	3.370 (4)	3.153 (2)	3.35	1.06
Br	3.249 (5)	3.064 (4)	3.46	1.13
I	3.127 (4)	3.074 (2)	3.61	1.17

**Table 3:** Mean NC⋯*X* contact lengths (Å) in 2,4,6-trihalophenyl isocyanides (*X*3NC)

*X*	*X*3NC	*r* [C + *X*]	[*r*/*X*3NC]
Cl	3.245 (3)	3.49	1.08
Br	3.151 (4)	3.60	1.14
I	3.106 (2)	3.75	1.21

**Table 4:** Experimental details

	I3CN	I3NC
Crystal data		
Chemical formula	C_7_H_2_I_3_N	C_7_H_2_I_3_N
*M*_r_	480.80	480.80
Crystal system, space group	Orthorhombic, *I* *m* *m* *a*	Orthorhombic, *I* *m* *m* *a*
Temperature (K)	100	100
*a*, *b*, *c* (Å)	7.0593 (4), 10.5346 (5), 13.0658 (6)	7.0552 (3), 10.4947 (5), 13.1557 (5)
*V* (Å^3^)	971.66 (8)	974.08 (7)
*Z*	4	4
Radiation type	Mo *K*α	Mo *K*α
μ (mm^−1^)	9.59	9.56
Crystal size (mm)	0.12 × 0.10 × 0.10	0.15 × 0.09 × 0.07

Data collection		
Diffractometer	Bruker VENTURE PHOTON-II	Bruker VENTURE PHOTON-II
Absorption correction	Multi-scan (*SADABS*; Sheldrick, 1996 ▸)	Multi-scan (*SADABS*; Sheldrick, 1996 ▸)
*T*_min_, *T*_max_	0.281, 0.344	0.251, 0.344
No. of measured, independent and observed [*I* > 2σ(*I*)] reflections	8436, 1303, 1261	13040, 1314, 1267
*R*_int_	0.024	0.026
(sin θ/λ)_max_ (Å^−1^)	0.835	0.834

Refinement		
*R*[*F* ^2^ > 2σ(*F* ^2^)], *wR*(*F* ^2^), *S*	0.013, 0.030, 1.18	0.011, 0.024, 1.14
No. of reflections	1303	1314
No. of parameters	40	40
H-atom treatment	H-atom parameters constrained	H-atom parameters constrained
Δρ_max_, Δρ_min_ (e Å^−3^)	0.89, −0.60	0.72, −0.48

Computer programs: *APEX3* (Bruker, 2012 ▸), *SAINT* (Bruker, 2012 ▸), *SHELXT2014* (Sheldrick, 2015*a* ▸), *SHELXL2014* (Sheldrick, 2015*b* ▸), *Mercury* (Macrae *et al.*, 2008 ▸), *publCIF* (Westrip, 2010 ▸).

## supplementary crystallographic information

### 2,4,6-Triiodobenzonitrile (I3CN) Crystal data

**Table:**

C_7_H_2_I_3_N	*D*_x_ = 3.287 Mg m^−^^3^
*M**_r_* = 480.80	Melting point: 517 K
Orthorhombic, *I**m**m**a*	Mo *K*α radiation, λ = 0.71073 Å
*a* = 7.0593 (4) Å	Cell parameters from 2721 reflections
*b* = 10.5346 (5) Å	θ = 2.5–36.4°
*c* = 13.0658 (6) Å	µ = 9.59 mm^−^^1^
*V* = 971.66 (8) Å^3^	*T* = 100 K
*Z* = 4	Square bipyramid, colorless
*F*(000) = 840	0.12 × 0.10 × 0.10 mm

### 2,4,6-Triiodobenzonitrile (I3CN) Data collection

**Table:**

Bruker VENTURE PHOTON-II diffractometer	1261 reflections with *I* > 2σ(*I*)
Radiation source: micro-focus	*R*_int_ = 0.024
φ and ω scans	θ_max_ = 36.4°, θ_min_ = 2.5°
Absorption correction: multi-scan (SADABS; Sheldrick, 1996)	*h* = −9→11
*T*_min_ = 0.281, *T*_max_ = 0.344	*k* = −17→17
8436 measured reflections	*l* = −21→21
1303 independent reflections	

### 2,4,6-Triiodobenzonitrile (I3CN) Refinement

**Table:**

Refinement on *F*^2^	Hydrogen site location: inferred from neighbouring sites
Least-squares matrix: full	H-atom parameters constrained
*R*[*F*^2^ > 2σ(*F*^2^)] = 0.013	*w* = 1/[σ^2^(*F*_o_^2^) + (0.0078*P*)^2^ + 1.2371*P*] where *P* = (*F*_o_^2^ + 2*F*_c_^2^)/3
*wR*(*F*^2^) = 0.030	(Δ/σ)_max_ = 0.001
*S* = 1.18	Δρ_max_ = 0.89 e Å^−^^3^
1303 reflections	Δρ_min_ = −0.60 e Å^−^^3^
40 parameters	Extinction correction: SHELXL2014 (Sheldrick, 2015b), Fc^*^=kFc[1+0.001xFc^2^λ^3^/sin(2θ)]^-1/4^
0 restraints	Extinction coefficient: 0.00199 (9)

### 2,4,6-Triiodobenzonitrile (I3CN) Special details

**Table:**

Geometry. All esds (except the esd in the dihedral angle between two l.s. planes) are estimated using the full covariance matrix. The cell esds are taken into account individually in the estimation of esds in distances, angles and torsion angles; correlations between esds in cell parameters are only used when they are defined by crystal symmetry. An approximate (isotropic) treatment of cell esds is used for estimating esds involving l.s. planes.

### 2,4,6-Triiodobenzonitrile (I3CN) Fractional atomic coordinates and isotropic or equivalent isotropic displacement parameters (Å^2^)

**Table:**

	*x*	*y*	*z*	*U*_iso_*/*U*_eq_	
I2	0.5000	0.46395 (2)	0.83491 (2)	0.01267 (4)	
I4	0.5000	0.7500	0.43429 (2)	0.01359 (4)	
N7	0.5000	0.7500	1.00506 (18)	0.0167 (4)	
C1	0.5000	0.7500	0.80692 (18)	0.0103 (4)	
C2	0.5000	0.63456 (15)	0.75302 (13)	0.0105 (3)	
C3	0.5000	0.63428 (16)	0.64671 (13)	0.0117 (3)	
H3	0.5000	0.5565	0.6100	0.014*	
C4	0.5000	0.7500	0.59458 (19)	0.0113 (4)	
C7	0.5000	0.7500	0.9170 (2)	0.0130 (4)	

### 2,4,6-Triiodobenzonitrile (I3CN) Atomic displacement parameters (Å^2^)

**Table:**

	*U*^11^	*U*^22^	*U*^33^	*U*^12^	*U*^13^	*U*^23^
I2	0.01764 (6)	0.00914 (5)	0.01122 (5)	0.000	0.000	0.00198 (3)
I4	0.01801 (8)	0.01463 (7)	0.00812 (6)	0.000	0.000	0.000
N7	0.0218 (11)	0.0137 (9)	0.0145 (9)	0.000	0.000	0.000
C1	0.0104 (9)	0.0113 (9)	0.0094 (8)	0.000	0.000	0.000
C2	0.0123 (6)	0.0088 (6)	0.0104 (6)	0.000	0.000	0.0017 (5)
C3	0.0157 (7)	0.0095 (6)	0.0099 (6)	0.000	0.000	0.0002 (5)
C4	0.0121 (9)	0.0115 (9)	0.0104 (9)	0.000	0.000	0.000
C7	0.0137 (10)	0.0120 (9)	0.0133 (10)	0.000	0.000	0.000

### 2,4,6-Triiodobenzonitrile (I3CN) Geometric parameters (Å, º)

**Table:**

I2—C2	2.0916 (16)	C1—C7	1.438 (3)
I4—C4	2.094 (2)	C2—C3	1.389 (2)
N7—C7	1.151 (3)	C3—C4	1.396 (2)
C1—C2	1.405 (2)	C3—H3	0.9500
C1—C2^i^	1.405 (2)	C4—C3^i^	1.396 (2)
			
C2—C1—C2^i^	119.9 (2)	C2—C3—H3	120.5
C2—C1—C7	120.07 (11)	C4—C3—H3	120.5
C2^i^—C1—C7	120.07 (11)	C3^i^—C4—C3	121.6 (2)
C3—C2—C1	120.19 (16)	C3^i^—C4—I4	119.19 (11)
C3—C2—I2	120.65 (12)	C3—C4—I4	119.19 (11)
C1—C2—I2	119.16 (13)	N7—C7—C1	180.0
C2—C3—C4	119.07 (16)		
			
C2^i^—C1—C2—C3	0.000 (1)	C1—C2—C3—C4	0.000 (1)
C7—C1—C2—C3	180.000 (1)	I2—C2—C3—C4	180.000 (1)
C2^i^—C1—C2—I2	180.000 (1)	C2—C3—C4—C3^i^	0.000 (1)
C7—C1—C2—I2	0.000 (1)	C2—C3—C4—I4	180.000 (1)

Symmetry code: (i) −*x*+1, −*y*+3/2, *z*.

### 2,4,6-Triiodophenyl isocyanide (I3NC) Crystal data

**Table:**

C_7_H_2_I_3_N	*D*_x_ = 3.279 Mg m^−^^3^
*M**_r_* = 480.80	Melting point: 467 K
Orthorhombic, *I**m**m**a*	Mo *K*α radiation, λ = 0.71073 Å
*a* = 7.0552 (3) Å	Cell parameters from 2833 reflections
*b* = 10.4947 (5) Å	θ = 2.5–36.3°
*c* = 13.1557 (5) Å	µ = 9.56 mm^−^^1^
*V* = 974.08 (7) Å^3^	*T* = 100 K
*Z* = 4	Block, colourless
*F*(000) = 840	0.14 × 0.09 × 0.07 mm

### 2,4,6-Triiodophenyl isocyanide (I3NC) Data collection

**Table:**

Bruker VENTURE PHOTON-II diffractometer	1267 reflections with *I* > 2σ(*I*)
Radiation source: micro-focus	*R*_int_ = 0.026
φ and ω scans	θ_max_ = 36.3°, θ_min_ = 2.5°
Absorption correction: multi-scan (SADABS; Sheldrick, 1996)	*h* = −11→11
*T*_min_ = 0.251, *T*_max_ = 0.344	*k* = −17→12
13040 measured reflections	*l* = −21→21
1314 independent reflections	

### 2,4,6-Triiodophenyl isocyanide (I3NC) Refinement

**Table:**

Refinement on *F*^2^	Hydrogen site location: inferred from neighbouring sites
Least-squares matrix: full	H-atom parameters constrained
*R*[*F*^2^ > 2σ(*F*^2^)] = 0.011	*w* = 1/[σ^2^(*F*_o_^2^) + (0.0044*P*)^2^ + 1.2499*P*] where *P* = (*F*_o_^2^ + 2*F*_c_^2^)/3
*wR*(*F*^2^) = 0.024	(Δ/σ)_max_ = 0.001
*S* = 1.14	Δρ_max_ = 0.72 e Å^−^^3^
1314 reflections	Δρ_min_ = −0.48 e Å^−^^3^
40 parameters	Extinction correction: SHELXL2014 (Sheldrick, 2015b), Fc^*^=kFc[1+0.001xFc^2^λ^3^/sin(2θ)]^-1/4^
0 restraints	Extinction coefficient: 0.00312 (10)

### 2,4,6-Triiodophenyl isocyanide (I3NC) Special details

**Table:**

Geometry. All esds (except the esd in the dihedral angle between two l.s. planes) are estimated using the full covariance matrix. The cell esds are taken into account individually in the estimation of esds in distances, angles and torsion angles; correlations between esds in cell parameters are only used when they are defined by crystal symmetry. An approximate (isotropic) treatment of cell esds is used for estimating esds involving l.s. planes.

### 2,4,6-Triiodophenyl isocyanide (I3NC) Fractional atomic coordinates and isotropic or equivalent isotropic displacement parameters (Å^2^)

**Table:**

	*x*	*y*	*z*	*U*_iso_*/*U*_eq_	
I12	0.5000	0.46223 (2)	0.83472 (2)	0.01190 (3)	
I14	0.5000	0.7500	0.43715 (2)	0.01245 (4)	
N17	0.5000	0.7500	0.91223 (14)	0.0120 (3)	
C11	0.5000	0.7500	0.80681 (15)	0.0098 (3)	
C12	0.5000	0.63389 (13)	0.75389 (11)	0.0102 (2)	
C13	0.5000	0.63411 (14)	0.64790 (11)	0.0112 (2)	
H13	0.5000	0.5561	0.6113	0.013*	
C14	0.5000	0.7500	0.59640 (15)	0.0108 (3)	
C17	0.5000	0.7500	1.00074 (17)	0.0150 (4)	

### 2,4,6-Triiodophenyl isocyanide (I3NC) Atomic displacement parameters (Å^2^)

**Table:**

	*U*^11^	*U*^22^	*U*^33^	*U*^12^	*U*^13^	*U*^23^
I12	0.01610 (5)	0.00910 (4)	0.01051 (4)	0.000	0.000	0.00184 (3)
I14	0.01561 (6)	0.01413 (6)	0.00761 (5)	0.000	0.000	0.000
N17	0.0134 (7)	0.0125 (7)	0.0102 (7)	0.000	0.000	0.000
C11	0.0106 (8)	0.0106 (7)	0.0081 (7)	0.000	0.000	0.000
C12	0.0121 (5)	0.0086 (5)	0.0098 (5)	0.000	0.000	0.0012 (4)
C13	0.0138 (6)	0.0096 (5)	0.0101 (5)	0.000	0.000	0.0000 (4)
C14	0.0124 (8)	0.0116 (8)	0.0086 (7)	0.000	0.000	0.000
C17	0.0174 (9)	0.0148 (9)	0.0127 (8)	0.000	0.000	0.000

### 2,4,6-Triiodophenyl isocyanide (I3NC) Geometric parameters (Å, º)

**Table:**

I12—C12	2.0920 (14)	C11—C12	1.4035 (17)
I14—C14	2.095 (2)	C12—C13	1.394 (2)
N17—C17	1.164 (3)	C13—C14	1.3922 (17)
N17—C11	1.387 (3)	C13—H13	0.9500
C11—C12^i^	1.4035 (17)	C14—C13^i^	1.3922 (17)
			
C17—N17—C11	180.0	C14—C13—C12	119.21 (14)
N17—C11—C12^i^	119.75 (9)	C14—C13—H13	120.4
N17—C11—C12	119.74 (9)	C12—C13—H13	120.4
C12^i^—C11—C12	120.51 (18)	C13^i^—C14—C13	121.76 (18)
C13—C12—C11	119.65 (13)	C13^i^—C14—I14	119.12 (9)
C13—C12—I12	120.65 (10)	C13—C14—I14	119.12 (9)
C11—C12—I12	119.70 (11)		
			
N17—C11—C12—C13	180.000 (1)	C11—C12—C13—C14	0.000 (1)
C12^i^—C11—C12—C13	0.000 (1)	I12—C12—C13—C14	180.000 (1)
N17—C11—C12—I12	0.000 (1)	C12—C13—C14—C13^i^	0.000 (1)
C12^i^—C11—C12—I12	180.000 (1)	C12—C13—C14—I14	180.000 (1)

Symmetry code: (i) −*x*+1, −*y*+3/2, *z*.
